# How is pain associated with pelvic mesh implants measured? Refinement of the construct and a scoping review of current assessment tools

**DOI:** 10.1186/s12905-022-01977-7

**Published:** 2022-09-30

**Authors:** Jennifer Todd, Jane E. Aspell, Michael C. Lee, Nikesh Thiruchelvam

**Affiliations:** 1 School of Psychology and Sport Science, Anglia Ruskin University, East Road, Cambridge, Cambridgeshire, CB1 1PT UK; 2 Centre for Psychological Medicine, Perdana University, Serdang, Malaysia; 3 Department of Medicine, University of Cambridge, Cambridge, UK; 4 Department of Anaesthesia, Cambridge University Hospitals NHS Foundation Trust, Cambridge, UK; 5 Department of Urology, Cambridge University Hospitals NHS Foundation Trust, Cambridge, UK

**Keywords:** Pain, Pelvic mesh, Assessment, Psychometric, Pelvic organ prolapse, Stress urinary incontinence

## Abstract

**Background:**

Recommendations for the management of pain related to pelvic mesh implants are still under development. One limitation that has impeded progress in this area is that mesh-related pain has not been consistently defined or measured. Here, we reviewed the ways in which pain associated with pelvic mesh implants has been measured, and mapped the ways in which these existing measures capture the construct.

**Methods:**

First, we reviewed existing accounts of the pain associated with pelvic mesh implants to develop a multifaceted construct definition, which includes aspects related to pain intensity, timing, body location, phenomenological qualities, impact/interference with daily living, and patient expectations and beliefs. Next, we reviewed the ways that the construct has been measured in the extant literature.

**Results:**

Within 333 eligible studies, 28 different assessments of pain associated with pelvic mesh were identified, and 61% of studies reported using more than one measurement tool. Questionnaire measures included measures designed to assess urological and/or pelvic symptoms, generic measures and unvalidated measures. We did not identify any validated questionnaire measures designed to assess pain associated with pelvic mesh implants. The phenomenological, location, and expectation/belief components of the construct were not captured well by the identified questionnaire measures, and there is no evidence that any of the identified measures have appropriate psychometric properties for the assessment of pain related to pelvic mesh implants.

**Conclusions:**

We recommend further qualitative research regarding women’s experiences of pelvic mesh-related pain assessment, and the development of a condition-specific patient reported outcome measure.

## Background

Research indicates between 6 to 12% of women who have undergone pelvic mesh surgery experienced complications directly related to the mesh implant [1–3], although the true number of cases remains unknown [4]. Documented complications include mesh exposure [5], mesh erosion [6, 7], injury to the bladder [5, 7], urinary retention [8], dyspareunia [6, 7, 9, 10], and pain [9–13].

A proportion of patients experience de novo chronic pelvic pain in the absence of abnormal mesh placement, mesh extrusion or erosion [12, 14, 15]. Chronic pain (i.e., lasting > 3 months) following pelvic mesh surgery has been estimated to occur in up to 30% of women [5, 16, 17], and prevalence estimates between 3 and 13% have been reported for de novo post-surgical pain [18–21]. However, it has been acknowledged that the prevalence of pain following pelvic mesh surgery has not been reliably estimated to date [4, 14, 16, 22], and recommendations for the management of mesh-related pain are still being developed [4, 14, 16, 22–24]. In some instances, mesh implants have been removed with pain as the primary indication (e.g., [9, 12]). Of those who underwent removal surgery for the primary indication of pain, symptom improvement or resolution was reported for between 45 and 86% of patients [9, 11–13, 25], while an estimated 9% reported no change, and an estimated 14% reported a worsening of symptoms [13]. However, further research on this topic is necessary [22], particularly regarding the longevity of pain resolution after mesh removal [25].

A key limitation that has impeded the progress of research on this topic is that the construct (i.e., “pain associated with pelvic mesh implants”) has not been consistently defined or measured. This, in turn, contributes to difficulties in estimating and comparing pain outcomes (e.g., for different implants, patient groups, or treatments). In the present work we sought to examine these issues by: (1) developing a working conceptualisation of what is meant by ‘pain associated with pelvic mesh implants’; (2) ascertaining the ways in which pain associated with pelvic mesh implants has been measured and quantified in published research; (3) mapping the ways in which these existing measures capture the construct, and; (4) reviewing evidence of psychometric properties of the included measures in samples of women with pelvic mesh implants. We addressed the first aim (development of a working conceptualisation of pain associated with pelvic mesh implants) via a narrative review of theoretical papers and qualitative accounts of pain associated with pelvic mesh implants. We addressed the second aim via a systematic scoping review, and we addressed the third and fourth aims via an in-depth review, which assessed the degree of coverage each measure provided of the construct, and evidence of the psychometric properties in samples of women with pelvic mesh implants.

More specifically, the aim of the initial narrative review was to identify components of the construct (i.e., ‘pain associated with pelvic mesh implants’) that should be considered when evaluating instruments that have been utilised to quantify pain associated with pelvic mesh implants. Given that a key theme from the Cumberledge report was that the patient voice was dismissed [4], the narrative review primarily focused on qualitative accounts of pain associated with pelvic mesh implants. Meanwhile, the aim of the scoping review was to identify the ways that pain associated with pelvic mesh implants has been measured in published research to date. Prior to conducting the search, we confirmed that no systematic or scoping reviews have been conducted on the same topic via a search of Medline, Embase, and Cochrane Database of Systematic Reviews. We used the scoping review framework developed by Peters and colleagues [33] to guide the scoping review. This method was chosen because it was particularly suitable for addressing our topic (i.e., the identification and assessment of assessments of pain associated with pelvic mesh implants used in clinical practice that have been described in published literature). Finally, we conducted a further in-depth review of the measures identified in the systematised search, mapping: (1) the extent to which each measure covers the breadth of the construct, and (2) evidence of the psychometric properties of each measure in samples of patients who have a pelvic mesh implant.

## Method

### Narrative review procedure

We searched for qualitative accounts of pain experiences from patients with pelvic mesh implants that have been published in peer-reviewed journals, and descriptions/definitions of pain associated with pelvic mesh implants that have been outlined in peer-reviewed journal articles. This search was generalised to publications that included any experiences of pain associated with pelvic mesh implants, and we did not distinguish between pain induced by abnormal mesh placement or abnormal evolution and pain with normal mesh placement and normal evolution [14]. The study authors (which include a consultant urologist, a consultant in pain medicine, and two cognitive neuroscientists with expertise in multisensory bodily representations), then reviewed and amalgamated the key findings to generate a working definition of the construct. In addition, we sought feedback on the method and results from a patient and public involvement (PPI) panel of ten women with pain related to pelvic mesh implants.

### Scoping review procedure

#### Inclusion criteria

##### Types of participants

The participants of interest for this study were adult women (aged 18 years or older) with a pelvic mesh implant or undergoing a surgery for a pelvic mesh implant.

##### Concept

Studies with at least one reported assessment of pain associated with a pelvic mesh in adult women (aged 18 years or older). Questionnaires used in the studies had to be in English language.

##### Context

The context for this review was healthcare settings with adult female patients undergoing any procedures or treatment associated with a pelvic mesh implant.

##### Sources

The review considered both experimental and epidemiological study designs, including randomised controlled trials, non-randomised controlled trials, quasi-experimental, before and after studies, prospective and retrospective cohort studies, case-studies, case–control studies, and analytical cross-sectional studies. To limit duplication and irrelevant material, exclusion criteria included articles in languages other than English, published abstracts, reviews or meta-analyses, commentaries, editorials, book chapters, and dissertations, studies examining men, children aged younger than 18 years, studies focusing on a pathology or procedure not listed in the search terms above, and studies that did not report an assessment of pain. Due to large volume of literature identified in preliminary searches, we did not conduct further searches for unpublished studies, clinical trial registries, or grey literature such as government reports, as this was unlikely to result in additional evidence in relation to our goal.

#### Search strategy

**Fig. 1:**
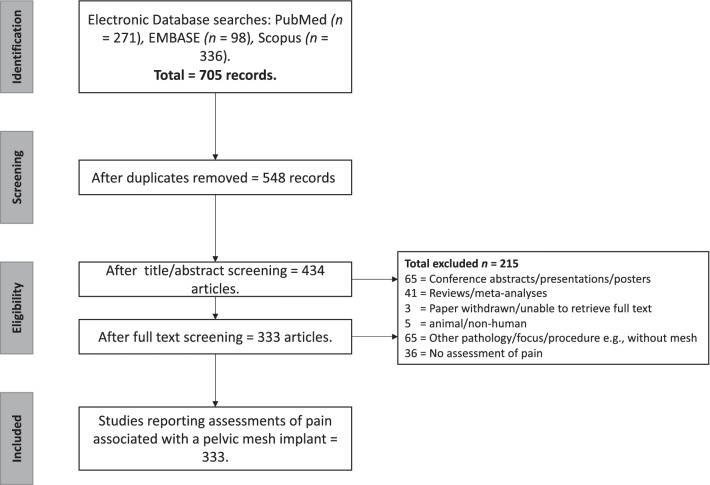
Search strategy

A two-step search strategy was utilised. The first step was an initial limited search of PubMed to explore the suitability of the search terms. Following this initial search, we analysed the text contained in the title and abstracts of retrieved papers. A second search was performed in December 2020, using PubMed, EMBASE, and Scopus. For each database, we searched article titles, abstracts and keywords using the following combination of search terms: (("Vaginal mesh" OR colporrhaphy OR "pelvic mesh" OR "pelvic floor reconstruct*" OR sacrocolpopexy OR sacropexy OR sacrohysteropexy OR "transvaginal mesh repair" OR "transobturator tape" OR "transvaginal tape") AND (pain* OR dyspareunia OR discomfort OR heaviness) AND (questionnaire* OR scale* OR rating* OR rated OR instrument* OR inventory OR inventories OR index OR score*) NOT (prostate OR men OR male OR "inguinal hernia" OR "femoral hernia" OR "umbilical hernia" OR breast OR cancer OR Schizophrenia OR child* OR adolescent* OR infant*)). We also manually applied search filters within each database to limit the records to English journal articles published in the last ten years, with human samples. Due to constraints on time and funding, articles were manually screened by J.T. for duplicates and for inclusion and exclusion criteria for final study selection (see Fig. 1). N.T. also screened a random 10% of the articles, and there were no discrepancies between the judgements of the two authors.

#### Data extraction

Data extraction focused on the names of assessments for pain associated with pelvic mesh implants. This review adhered to PRISMA guidelines for reporting Scoping reviews. Once the questionnaires were identified from the included studies, their development and validation studies were procured for further data extraction. Key information extracted included: domains assessed, number of items, structure of the measure, response scale type, and evidence of psychometric properties in samples of women with a pelvic mesh implant.

### In-depth review of identified measures

#### Conceptual review

To allow meaningful descriptive analyses and identification of research gaps, the identified pain assessments were mapped according to the components of the construct (i.e., pain associated with pelvic mesh implants) that were delineated in Table 1. We also used descriptive characteristics to portray the number of times each measure has been used within the dataset. For non-questionnaire measures (physical exam/patient report), we documented whether this was used as an exclusive measure, or whether this was supplementary to one of the other identified measures. We also documented the total number of studies using more than one of the measures. The conceptual review was undertaken by J.T. The remaining authors reviewed the method and findings.

**Table 1:** Components of pain associated with pelvic mesh implants illustrated by examples from published qualitative research and reviews

Reference and study type (organised alphabetically by first-author surname)	Components of pain associated with pelvic mesh implants					
	Timing (Onset/duration/ Frequency)	Intensity	Location	Phenomenological qualities	Impact/interference	Patient expectations/beliefs
Brown [27]—Qualitative	“When I was discharged I was still in the same amount of pain” “…the acute and chronic pain and the disabilities that I now live with”	“I am not sure how much more my body and spirit can take”		“It felt like when I was walking it would feel like I had barbed wire in me rubbing…”	*Emotional*: “I am not suicidal as such—I just don’t have the courage to do that—but I would rather no longer be here.” “In my darkest times I have thought about ending my life” *QOL*:** “**All of the things I could do are off the table now” “I feel it is the grief of losing my ‘pre-mesh’ life that brings me down the most” *Relationships*: “The problems caused by mesh for me has almost felt like a life sentence of home detention, it has been so isolating.”	“I did not need all of that surgery, I did not, probably did not need the mesh put in, there was other alternatives that he [my surgeon] never even offered me.” “It is not the life I envisioned for myself before I had the surgery. When the mesh was removed I had hoped there would be a big improvement, that my life would change, but it didn’t happen.”
Cadish et al. [28]—mixed methods	Postoperative onset pain highly prevalent at 2 weeks but decreased dramatically at 6 weeks	Mostly patients with pain reported that it was mild in severity. A minority reported severe postoperative-onset pain	Hip, leg, groin, incision site. A large proportion reported pain at more than one site			
Dunn et al. [29]—Qualitative	"I hurt all the time, all day, every day. I have not woken up pain-free since the surgery.”			“Like being ripped apart from the inside”	*Physical/QOL*: "It has destroyed my life. I cannot drive, I cannot travel, I cannot watch movies, I cannot wear heels; I can't drive my car because it's painful. My life has totally changed." *Emotional*: "I hope the pain and ache goes away, but I'm told it will likely not, which is depressing but not surprising." *Sexual/relationships:* "It took a big toll on my marriage as we were unable to be intimate for a year and a half.” *QOL*: "I've used up all of my sick pay and much of vacation."	‘‘He [the original surgeon] was irresponsible, he did not explain to me any consequences, he was dishonest, he said the surgery would be easy for me, I was so much worse.’’ ‘‘I wish I had never had it done. The doctor who placed it was supposed to be a good doctor, but it really messed things up and made my life miserable for a while.” ‘‘I was thinking she’d be able to take all the mesh out, but I wonder if the remaining piece is where the pain is. [I’m] still worried about remaining mesh.’’ “The mesh is balled up and I think it affects my bowel movements.”
Izett-Kay et al. [30] —Qualitative	“I am in pain every day since the operation” “I experienced a great deal of pain immediately after my op and my recovery took much longer than suggested”		“Lower back pain and heaviness in the vaginal area” A variety of codes for anatomical locations	“Heaviness”	*Physical*: “I don’t undertake heavy lifting now”	“I feel that [my pain] is because of the mesh but visits to a doctor and consultant have not confirmed, but not diagnosed anything else.” “What research was carried out on this vaginal mesh?” “I experienced a great deal of pain immediately after my op and my recovery took much longer than suggested so I think expectations should be adjusted”
Lee et al. [22]—Theory/review	Assessment should include duration of pain	Assessment should include severity of pain “Oftentimes the patient’s pain may be so severe that a detailed pelvic examination is impossible.”	Assessment should include location of pain and site of radiation Typically located in pelvic region, vaginal, or buttocks	Assessment should include nature and quality of pain	*Sex/relationship:* Dyspareunia *Physical/QOL*: Assessment should include aggravating and relieving factors	
Roos et al. [31]—Mixed methods			Vaginal	“[During sexual penetration] it seems that there is an ending, whereas before there didn't seem to be. You seem to hit something. (…) And the feeling that it was going to break, does that make sense? It feels as if it was, it was stretching.”	*Sex/relationships:* “Sometimes it makes you not want to do it. If you, if you can feel that it's prolapsed any way during the course of the day, and if I can feel that it's hanging right down, yeah you do feel that you don't want to do it sometimes, you just can't be bothered, because you know that it's already uncomfortable, so all that's going do is make it worse.”	“Because, you know, I was told that it would be quite tight to start with. Why don't I feel that it is? (…) I suppose I was expecting things to be a bit tighter than they are.”
Toozs-Hobson et al. [24]—Theory/review	*Patterns of pain*: Immediate onset Delayed Presentation (6 weeks to 3 months after surgery) Longer term delayed presentation (> 6 months after surgery) Late presentation (years after mesh insertion)					
Uberoi et al. [32]—Qualitative		“Just unbelievable pain”	Pelvic		*QOL*: “It stops your life, you're in pain, you're stopped” *Physical*: “It hurts to sit here”	“Nothing was mentioned about being in pain”

QOL = Quality of life. Quotations are all from women with pelvic mesh implants as reported in published qualitative studies, see study references

#### Psychometric review

We examined existing evidence of the psychometric properties for each of the identified questionnaire measures in samples with pelvic mesh implants. We limited the psychometric appraisal to aspects of reliability (internal consistency and test–retest reliability) and validity (convergent validity, minimally important clinical difference, and responsiveness). Internal consistency reliability refers to the extent to which items on a measure all tap the same construct. For the measures in this review, internal consistency was estimated using Cronbach’s α coefficient, (values ≥ 0.80 have been deemed as acceptable; [34, 35]. Test–retest reliability refers to the level of agreement between scores from the same measure at two separate timepoints. In the studies included within this review, test–retest reliability was typically reported using intraclass correlation coefficients (ICCs), with higher values indicative of greater reliability [36, 37]. Convergent validity refers to the extent to which scores on a measure are correlated with scores on other theoretically similar constructs (i.e., whether they are capturing the same construct [38]. Minimally important difference and responsiveness are related indices; responsiveness refers to a measure’s ability to detect changes in a respondent’s condition, and minimally important difference refers to the lowest change in score required to reflect a meaningful change in patient condition. Minimally important change can be estimated in numerous ways, including anchor-based and distribution-based methods, with a combined approach recommended [39]. The psychometric review was undertaken by J.T. The remaining authors reviewed the method and findings.

## Results

### Narrative review outcomes: definition of the construct

As described in Sect. 2.1, we integrated existing qualitative and theoretical accounts of the pain associated with pelvic mesh implants, which this informed the development of a multidimensional construct definition, with feedback from a PPI panel. The key findings from the narrative review are summarised in Table 1. Altogether, we suggest that pain associated with pelvic mesh implants is a multifaceted construct, with six key components: (1) timing, which includes the onset, duration, and frequency of pain in relation to pelvic mesh implantation; (2) the sensory intensity of pain (i.e., the subjective characterisation of how strongly pain is felt); (3) the bodily location of pain (i.e., where in the body the pain is felt); (4) other phenomenological qualities of pain (i.e., how pain feels, including affective-motivational aspects such as how unpleasant pain feels; see [26]); (5) the extent to which pain or protective behaviours impact upon or interfere with aspects of daily living; and, (6) patient expectations and beliefs, which include a priori expectations about the surgery and the mesh implant, as well as post-surgical beliefs about the predominant origin or cause of the patient’s pain, and the future nature of the patient’s pain. The impact/interference component (5) can be further categorised into four sub-components that may be affected by pain: physical functioning, sexual functioning/relationships, emotional functioning/mental health, and quality of life.

### Summary of the questionnaire measures identified

The search led to the identification of 333 eligible studies that have reported pain associated with pelvic mesh implants (see Fig. 1; the data extraction process for the full search results and screened findings are available here: https://figshare.com/s/88d23a55f7cad5833856). Within the 333 articles, 28 different assessments of pain associated with pelvic mesh were identified, as summarised in Table 2, and 204 studies (61%) reported using more than one measurement tool. We grouped “Physical exam/oral report” as one category, and also grouped “novel measure/measure with no psychometric validation” as one category. Questionnaire measures were reported independently if we were able to identify at least one published psychometric evaluation of the measure in any sample (including samples without a pelvic mesh implant).

**Table 2:** A summary of the measures used to quantify pain related to pelvic mesh implants

Name of measure	Number of studies using measure	Components of pain related to pelvic mesh implants covered by each measure					
		Timing (Onset/duration/ frequency of pain)	Intensity	Impact/interference*	Location**	Phenomenological qualities	Patient expectations or beliefs
Analgesia amount/length of time used	25	x	x				
Anatomical diagram for patient completion (non-standardised)	2				x		
Brief pain inventory	1		x	1, 2, 3, 4	x		
Bristol Female lower urinary tract symptoms questionnaire	1	x		2, 4	x	x	
Clavien-Dindo classification	17		x				
Diary of symptoms	5	x	x		x		
Electronic personal assessment questionnaire—pelvic floor	2	x		1, 2, 3, 4	x	x	
EQ-5D-5L	8		x	1, 3, 4			
Female genitourinary pain index	1	x	x	1, 2, 3, 4	x	x	
Female sexual function index	23	x	x	2		x	
International consultation on incontinence questionnaire vaginal symptoms	28	x		2, 4	x	x	
International urogynaecology association (IUGA) complication grades	19	x		1, 2	x		
King’s health questionnaire	12		x	1, 2, 3, 4	x		
McGill pain questionnaire	2		x	1, 2		x	
Novel measures/measures with no psychometric information***	28	N/A	N/A	N/A	N/A	N/A	N/A
Numeric rating scale for pain	15		x				
Pain catastrophizing scale	1		x	3			x
Patient global impression of improvement	56	x	x	1, 2, 3, 4			
Pelvic Floor distress inventory (PFDI; or the short form PFDI-20)	68	x		1	x	x	
Colorectal-anal distress inventory as standalone subscale	2	x		1			
Urinary distress inventory as standalone subscale	38	x		1	x		
Pelvic floor impact questionnaire (PFIQ; or the short form PFIQ-7)	44	x		1, 3, 4	x		
Pelvic organ prolapse–urinary incontinence sexual Function questionnaire (PISQ; or the short form PISQ-12, or revised PISQ-IR)	62	x		2		x	
Pelvic organ prolapse symptom score	2	x		1, 4	x	x	
Pelvic pain and urgency/frequency patient symptom scale	1	x	x	2, 4	x		
Physical exam and/or oral report as an exclusive measure***	70	N/A	N/A	N/A	N/A	N/A	N/A
Physical exam and/or oral report in addition to self-report measures***	100	N/A	N/A	N/A	N/A	N/A	N/A
Prolapse quality of life questionnaire	10	x		1, 2, 3, 4		x	
Short-form health survey (SF-36/SF-12)	13	x	x	1, 3, 4			
Visual analogue scale for pain	70		x				
Wong-Baker FACES scale	3		x				

*1 = Physical functioning, 2 = Sexual functioning/relationships, 3 = Emotional functioning/mental health, 4 = Other aspect of quality of life

**This box is marked if the items in the measure examined pain in a specific bodily location (e.g., Do you experience pain in your lower abdomen?) or if the measure has an open-ended question (or body map) regarding the location of pain

***Due to the breadth of different measures within these categories, it was not possible to accurately portray the components of mesh pain covered by the measures within Table 1. Full bibliographic information relating to each of these studies is available at https://figshare.com/s/88d23a55f7cad5833856

The identified measures can be broadly grouped into three categories: measures designed to assess urological and or/pelvic symptoms, generic measures (including measures validated across numerous other pain disorders), and unvalidated measures (including novel measures, or novel adaptations of measures).

#### Questionnaire measures designed to assess urological and/or pelvic symptoms

##### The Bristol female lower urinary tract symptoms questionnaire (BFLUTS)

The BFLUTS is a 34-item questionnaire measure that was designed to characterise symptoms in the female lower urinary tract, particularly urinary incontinence [40]. The BFLUTS has three sections: symptom severity (19 items), sexual function (4 items), and quality of life (11 items). Items pertaining specifically to pain include questions regarding burning sensation during urination, bladder pain, pain or discomfort because of a dry vagina, and pain during sexual intercourse. Most items are divided into two, with the first section quantifying the severity of the symptom and the second section quantifying the extent to which the symptom causes “bother”. A scored short form (the BFLUTS-SF, 19 items) was developed more recently [41], where summed scores can be computed separately for urinary symptoms (which has three factors: incontinence, voiding, and filling), sexual functional, and quality of life. In the BFLUTS-SF, only one of the items pertaining to pain was retained, specifically the question regarding frequency of bladder pain.

##### The electronic personal assessment questionnaire—pelvic floor (ePAQ-PF)

The ePAQ-PF a 132-item web-based questionnaire that was designed to enhance patient communication regarding pelvic floor disorders [42, 43]. The ePAQ-PF assesses the frequency and impact of pelvic floor symptoms across four domains: urinary (35 items), bowel (33 items), vaginal (22 items), and sexual (28 items) [42, 44]. Screening questions at the beginning of each questionnaire domain are used to streamline the items, so that not all respondents will be presented with the full item set. Within the four ePAQ-PF dimensions, there are 19 scored domains, three of which are focused on pain (‘pain’ within the urinary dimension; ‘pain and sensation’ within the vaginal dimension; and ‘dyspareunia’ within the sexual dimension [42]). Symptom items are scored between 0 (indicating best health status) and 3 (indicating worst health status). Each domain is scored by dividing the sum of all item scores by the total possible item score, multiplied by 100. The result is a score between 0 and 100, where 100 indicates the worst possible health status [42]. When a symptom item is affirmed, impact items are presented to examine the extent to which the symptom causes “bother”. Bother responses are scored on a four-point scale ranging from 0 (*not a problem*) to 3 (*a serious problem*). The largest bother score from any symptom in the domain is presented at the overall bother score for the domain, so that the total bother score ranges from 0 to 3. Finally, the ePAQ-PF includes a free-text question, which allows respondents to communicate the outcomes that they hope to achieve from any help, advice or treatment given [45].

##### The female genitourinary pain index (fGUPI)

The 15-item fGUPI was developed by modifying the National Institutes of Health Chronic Prostatitis Symptom Index so that it could be used to assess interstitial cystitis or painful bladder syndrome in women [46]. Male-specific items were adjusted so that they could capture symptoms that have commonly been reported in women with interstitial cystitis or painful bladder syndrome, including pain at the entrance to the vagina, pain in the vagina, pain in the urethra, and pain during or after sexual intercourse. The fGUPI is divided into three subscales: pain (10 items), urinary symptoms (2 items), and quality of life (3 items), and response scales vary across items, including binary *yes/no* responses to indicate the presence of pain in various locations (e.g., vagina, urethra) or accompanying various actions (e.g., during urination or sexual intercourse), a numeric rating scale to indicate the pain intensity, and Likert scales to indicate pain frequency. Total fGUPI scores are calculated by summing responses from all items, and range from 0 (indicative of no symptoms/pain) to 45 (indicative of severe symptoms/pain).

##### Female sexual function index (FSFI)

The 19-item FSFI was developed to measure the multidimensional nature of female sexual function in women across wide age range, including post-menopausal women [47]. Specifically, sexual function is assessed across six domains: desire (2 items), subjective arousal (4 items), lubrication (4 items), orgasm (3 items), satisfaction (3 items), and (3 items). Items pertaining to specifically to pain include questions regarding the frequency and level of pain or discomfort during or following vaginal penetration. For all items, responses are given on 6-point Likert scales ranging from 0 to 5. Scores for each domain are computed by multiplying the sum of scores on the domain by a weighted factor for each domain (e.g., total pain score = [Item 17 + 18 + 19] × 0.4), with domain scores ranging from 0 to 6 [47]. Global FSFI scores are computed by summing the scores from each domain.

##### International consultation on incontinence questionnaire-vaginal symptoms (ICIQ-VS)

The 14-item ICIQ-VS was designed to assess the symptoms and impact of pelvic organ prolapse in adult women across primary and secondary care settings [48]. The ICIQ-VS comprises two factors: vaginal symptoms (9 items) and sexual matters (4 items), as well as a single item assessing the extent to which vaginal symptoms interfere with overall quality of life. Items pertaining to specifically to pain include questions regarding dragging pain in the lower abdomen, soreness in the vagina, and feeling that the vagina is too dry or tight. Items are responded to using 4 and 5-point Likert scales ranging from 0 to 3 or 4. Each item is also accompanied by a 10-point numeric rating scale which is used to quantify the degree of “bother” caused by the symptom. Scores on the Vaginal Symptoms subscale range from 0–53, with higher scores indicating greater symptomology. Scores on the Sexual Matters subscale range from 0 to 58, with higher scores indicating greater degree of impact on sexual function. Notably, Item 9 (“vagina too tight”) is not included in the scoring system, but remains within the questionnaire to detect for over-narrowing of the vagina following surgical interventions [48].

##### The king’s health questionnaire (KHQ)

The 32-item KHQ was designed as a disease-specific QOL measure for women who experience urinary incontinence [49]. The questionnaire has three sections: a symptom severity Sect. (11 items), a general health and incontinence impact Sect. (2 items), and a QOL Sect. (19 items). The QOL section is further divided into seven domains, to examine whether the respondent’s “bladder problem” causes role limitations, physical limitations, social limitations, personal relationships, emotional problems, sleep/energy disturbance, and an index of strategies used to for coping with symptom severity. One item within the symptom severity index pertains specifically to “bladder pain”. Items are responded to using Likert scales that range from 3-point anchors (the symptom severity index anchors range from *A little* to *A lot*) to 4-point anchors (the QOL index anchors range from *Not at all* to *A lot*, with the additional option to indicate *Not applicable* for items related to personal relationships). Scores from the items in each domain are converted so that each domain score ranges from 0 (best possible health status) to 100 (worst possible health status), with a global score calculable across domains [50]. The symptom severity section can also be scored, with total scores ranging from 0 (best possible health status) to 30 (worst possible health status) [50].

##### The pelvic floor distress inventory (PFDI; or the short form PFDI-20)

The 46-item PFDI was designed to assess the level of distress caused by symptoms for women with pelvic floor dysfunction [51]. Disorders of the pelvic floor include several interrelated conditions, such as pelvic organ prolapse, urinary incontinence, faecal incontinence and voiding dysfunction [51]. The PFDI has three scales: the Urinary Distress Inventory (UDI, 28 items), the Pelvic Organ Prolapse Distress Inventory (POPDI, 16 items), and the Colorectal-anal Distress Inventory (CRADI, 17 items). A short form of the PFDI (the PFDI-20) was developed to reduce participant burden [52, 53], and this version was most commonly identified in our systematic search. The PFDI-20 maintains the structure of the full PFDI but has fewer items (UDI: 6 items, POPDI: 6 items, CRADI: 8 items). Four PFDI-20 items pertain specifically to pain, examining: pressure in the lower abdomen (POPDI-6), heaviness or dullness in the pelvic area (POPDI-6), pain when passing stools (CRADI-8), and pain or discomfort in the lower abdomen or genital region (UDI-6). Additional items pertaining to pain in the full PFDI examine pelvic discomfort during exertion (POPDI), pain in the abdomen or lower back when straining (POPDI, CRADI), pain or burning when urinating, or as the bladder fills (UDI), and abdominal pain prior to bowel movements.

PFDI-20 items are rated for the degree to which the symptom causes bother on a 5-point Likert scale ranging from 0 (*not applicable*), to 1 (*not at all*), to 4 (*quite a bit*). Summary scores for each scale are obtained by computing the mean of all items on the scale and multiplying this value by 25; possible scores range from 0 (best possible health state) to 100 (worst possible health state). A PFDI-20 summary score is obtained by summing the scores from the UDI-6, POPDI-6, and CRADI-8; possible scores range from 0 (best possible health state) to 300 (worst possible health state).

##### The pelvic floor impact questionnaire (PFIQ; or the short form PFIQ-7)

The 93-item PFIQ was developed alongside the PFDI to quantify the impact that pelvic floor disorders have on quality of life including assessment of physical, emotional, and social limitations [51]. Like the PFDI, the PFIQ has three scales: the Urinary Impact Questionnaire (UIQ, 31 items), the Pelvic Organ Prolapse Impact Questionnaire (POPIQ, 31 items), and the Colorectal-Anal Impact Questionnaire (CRAIQ, 31 items). None of the PFIQ items directly reference pain. Rather, the items examine if “symptoms or conditions” related to the bladder or urine (UIQ), bowel or rectum (CRAIQ), and vagina or pelvis affect aspects of daily life (the ability to do household chores such as cooking, housecleaning, and laundry). Accordingly, the PFIQ can encompass pain if it is relevant to the respondent.

A short form of the PFIQ (the PFIQ-7) was developed to reduce participant burden [52, 53], and this version was most commonly identified in our systematic search. The PFIQ-7 maintains the structure of the full PFIQ but has fewer items (UIQ-7: 7 items, POPIQ-7: 7 items, CRAIQ-7: 7 items). PFIQ-7 items are rated on a 4-point Likert scale ranging from 0 (*not at all*) to 3 (*quite a bit*). Summary scores for each scale are obtained by computing the mean of all items on the scale and multiplying this value by (100/3); possible scores range from 0 (best possible health state) to 100 (worst possible health state). A PFIQ-7 summary score is obtained by summing the scores from the UIQ-7, POPIQ-7 and CRAIQ-7; possible scores range from 0 (best possible health state) to 300 (worst possible health state).

##### The pelvic organ prolapse–urinary incontinence sexual function questionnaire (PISQ; or the short form PISQ-12, or revised PISQ-IR)

The 31-item PISQ was designed to evaluated sexual function in women with pelvic organ prolapse or urinary incontinence [54]. The PISQ has three domains: Physical (15 items), Behavioural/Emotive (10 items), and Partner Related (6 items). A short form of the PISQ (the PISQ-12, 12 items) was developed to reduce participant burden [54], and this version was the most commonly identified in our systematic search. The PISQ-12 reflects the content of the PISQ factors, but has fewer items and is unidimensional [54]. More recently, the International Urogynaecology Association revised the PISQ (the PISQ-IR, 42 items) to make it suitable for sexually active and inactive women [55]. One item from the PISQ-12 and the PISQ-IR pertains to pain (“Do you feel pain during sexual intercourse?”). There are no additional items directly referencing pain in the full PISQ, but three items reference avoidance of sexual intercourse due to vaginal “tightness” or “dryness”.

PISQ-12 items are rated on a 5-point Likert scale, ranging from 0 (*never*) to 4 (*always*). Scores can be reported as the summed total of the 12 items, or on an item basis [54]. In our strategic search, Item 5 from the PISQ-12 (“Do you feel pain during sexual intercourse?”) was commonly reported as an indication of dyspareunia.

##### The pelvic organ prolapse symptom score (POP-SS)

The 8-item POP-SS [56, 57] was designed to concisely capture the presence and extent of key pelvic organ prolapse symptoms. Three POP-SS items pertain to pain, examining the presence of: an uncomfortable feeling or pain in the vagina which is worse when standing; heaviness or a dragging feeling in the lower abdomen; and heaviness or a dragging feeling in the lower back. POP-SS items are rated on a 5-point Likert scale ranging from 0 (*never*) to 4 (*all of the time*), and scores are computed by summing scores from the first seven items. The final item (Item 8) is used to indicate which of the previous items causes the most “bother” and is not included in total POP-SS scores.

##### The pelvic pain and urgency/frequency patient symptom scale (PUF)

The 11-item PUF was designed to capture key symptoms of interstitial cystitis, including pelvic pain, decreased sexual functioning, and urinary urgency and frequency [58]. Seven PUF items assess symptoms, and four items assess the degree of bother caused by symptoms. Items pertaining to pain include assessments of pain frequency in the bladder or pelvis (including vagina, lower abdomen, and urethra), pain intensity, and the frequency of pain during or after sexual intercourse. Two items also examine the degree of “bother” caused by the pain. Most items are rated on a 4-point Likert scale, ranging from 0 (*never*) to 3 (*always*). “Symptom” and “bother” scores are computed by summing the scores from each item set, and a total score is computed by summing scores from the total item set. Possible scores range from 0 (best possible health state) to 35 (worst possible health state).

##### The prolapse quality of life questionnaire (P-QOL)

The P-QOL was designed to assess the severity of pelvic organ prolapse symptoms, and their impact on quality of life [59]. The P-QOL is divided into two sections. The first section examines the severity of urinary, vaginal and colorectal symptoms (18 items). This section also includes two questions regarding general health and quality of life. The second Sect. (18 items) examines the extent to which pelvic organ prolapse symptoms limit quality of life domains, including role limitations (2 items), physical and social limitations (4 items), impact on relationships (3 items), emotional impact (3 items), impact upon sleep and energy (2 items), and any severity measures employed to ease symptoms (4 items). Items pertaining to pain include assessment of heaviness or a dragging feeling from the vagina or lower abdomen; discomfort in the vagina which is worse when standing and relieved by lying down; lower backache which worsens with vaginal discomfort; and, pain or discomfort due to the prolapse.

P-QOL items are responded to using a 4-point Likert scale, ranging from *none* to *a lot*, with the option to indicate *not applicable* for the relationship items. Digesu, Khullar, and colleagues [59] (see also [60]) state that scores in each domain range between 0 (good quality of life) and 100 (greatly impaired quality of life).

#### Generic questionnaire measures

##### The brief pain inventory (BPI)

The BPI is a 15-item questionnaire measure that was designed to assess the pain experiences of clinical groups; however, it is now used widely [61]. The measure divides pain experience into two components: pain severity and interference. The severity index has a numeric rating scale, and respondents are asked to quantify the target pain as it was experienced at its “worst”, “least”, “on average” over the previous 24 h. Respondents are also asked to quantify the severity of pain experienced in the present moment. The severity index can be scored as a mean of the four items [62–64]. The interference index has an 11-point numeric rating scale, and respondents are asked to quantify the extent to which pain interferes with seven aspects of functioning, which include relationships, enjoyment of life, mood/emotional state, sleep, walking, work, and general activity [61]. The BPI also includes a bodily map where respondents mark locations where pain is experienced, and the location where the “worst” pain is experienced; a preliminary screening question examining whether the respondent has experienced pain on the day of completion; and additional questions about the extent of relief provided by medication or other treatment. These items are not typically reported in the BPI scores [61].

##### The EQ-5D-5L

The EQ-5D-5L is a generic measure of health-related quality of life [65]. The first section of the measure comprises five items, each covering a different domain (mobility, self-care, usual activities, anxiety/depression). For each item, participants indicate one of five response levels to reflect their present health state, where 1 indicates no problem and 5 indicates extreme problems (e.g., pain: 1 = *No pain discomfort*, 5 = *I have extreme pain or discomfort*). To ensure that each dimension is reflected in the final score, a 5-digit code is used to reflect the respondent’s health state, which each number corresponding to the response from each of the five dimensions (e.g., 1-1-3-2-2) [66]. It is also possible to assign each health state a summary index score based on national standard value sets for each the health state (e.g., [67]). Health state index scores generally range from 0 (where 0 is the value of a health state equivalent to dead) to 1 (the value of full health). In the second section of the ED-5D-5L, a visual analogue scale (VAS) is used to quantify the respondent’s subjective overall health rating, with scores ranging from 0 (*worst health you can imagine*) to 100 (*best health you can imagine*).

##### The McGill pain questionnaire (MPQ)

The MPQ [68] is a multidimensional pain questionnaire that was designed to facilitate communication regarding perceptual aspects of pain experience between patients and clinicians [69]. The questionnaire is comprised of 78 descriptors, which are grouped into word sets of two to six words, according to indications of pain intensity (e.g., word set 2: ‘shooting’ indicates more pain than ‘flashing’, which, in turn, indicates more pain than ‘jumping’). The word sets fall into four overarching categories: sensory (10 word sets), affective (5 word sets), evaluative (1 word set), and miscellaneous (4 word sets). Three indices can be computed from this [69]: (1) The number of words chosen; (2) the position of the word in each set indicates a ranked value (e.g., ‘jumping’ = 1, ‘flashing’ = 2, ‘shooting’ = 3), and pain rating indices are computed by summing the ranked values for all selected words in each category; and (3) the total score is computed by summing all ranked values.

The MPQ also includes a present pain intensity scale, which ranges from 0 (*no pain*) to 5 (*excruciating*) with descriptors for duration and frequency (e.g., ‘brief’, ‘rhythmic’, ‘constant’). Finally, the MPQ includes a body map presented from ventral and dorsal planes, so that pain distribution can be mapped with markers for internal and external locations.

##### The numeric rating scale (NRS) for pain

The NRS for pain is a widely-used single-item measure of pain intensity in adults (for a related overview, see [70]). Although many iterations of the NRS have been reported previously [70], the vast majority of studies identified in our systematic search used an 11-point NRS, which was presented verbally. In terms of scoring, zero typically represents “no pain”, and the number ten typically represents “pain as bad as you can imagine”/“worst pain imaginable”.

##### The pain catastrophizing scale (PCS)

The PCS is a 13-item measure of catastrophising (i.e., “an exaggerated negative mental set brought to bear during actual or anticipated painful experience” [71]: p.52), that was designed for use in clinical and non-clinical populations [72]. As such, it is primarily a measure of pain-related thoughts and beliefs (e.g., “When I am in pain it’s terrible and I think it’s never going to get any better”). The PCS has a higher-order factor structure, whereby the global construct (general catastrophising) is measured by three related dimensions: rumination (4 items), magnification (3 items), and helplessness (6 items) [73]. Each PCS item is rated on a 5-point scale, which ranges from 0 (*not at all*) to 4 (*all the time*). The PCS total score is computed by summing scores from all 13 items, with possible scores ranging from 0 to 52. Subscale scores are computed by summing responses from the items on each subscale.

##### The patient global impression of improvement (PGI)

The PGI is a single-item measure of the respondent’s subjective global impression of improvement, which was used with reference to the respondent’s change in condition before and after surgery in the studies identified in our systematic search (e.g., “What best describes how your post-operative condition is now, compared to how it was before you had surgery?”). Accordingly, the PGI does not directly pertain to pain, but might encompass pain if relevant to the respondent. The PGI has seven response categories, ranging from 1 (*very much better*), to 7 (*very much worse*) [74].

##### The short-form health survey (SF-36; or the abridged SF-12)

The SF-36 is a generic measure of health-related quality of life [75]. The SF-36 is comprised of 36 items, which examine components of physical health (four subdomains) and mental health (four subdomains) [75]. The physical health subdomains include physical functioning (10 items), role limitations due to physical health (4 items), bodily pain (2 items), and general health perceptions (5 items). The mental health subdomains include energy/fatigue (4 items), role limitations due to emotional problems (3 items), social functioning (2 items), and general mental health perceptions (5 items). The SF-36 also includes an additional health transition item (which asks the respondent to compare their current health state to their health state one year ago) which is not included in SF-36 summary scores. The two pain items examine the intensity of pain experienced and the extent to which pain interfered normal work over the preceding four-week period. A 12-item short-form of the SF-36 (the SF-12) contains only the pain interference item [76, 77].

SF-36 items are responded to using Likert scales with a varying number of degrees, with the majority use a 5-point response scale. Scoring the SF-36 is a complex process, involving the transformation and aggregation of raw scores. Final SF-36 scores can be computed for the eight subscales and two summary scales, ranging from 0 (poorest health state) to 100 (best health state) [75]. Scores can then be interpreted against standardised population norms [75].

##### The visual analogue scale for pain (VAS)

The VAS for pain is a graphical single-item measure of pain intensity in adults (for a related overview, see [69]). The VAS is comprised of a horizontal or vertical line—usually of a standardised 100 mm length—anchored by two opposing pain intensity descriptors (e.g., “no pain” versus “worst pain imaginable”). Although many iterations of the VAS have been reported previously [70], most studies identified in our systematic search collapsed the continuous measure to an 11-point scoring system where zero typically represented “no pain”, and the number ten typically represented “pain as bad as you can imagine”/ “worst pain imaginable”. A minority of studies scored the VAS using a range from 0 to 100 mm.

##### The Wong–Baker FACES scale (WBS)

The WBS was developed to facilitate assessments of pain intensity in children [78]. The WBS is a single-item, 6-point ordinal scale, ranging from 0 (*no hurt*) to 5 (*hurts worst*). Each scale point is accompanied by an illustration of a face, which is pictured smiling at point 0, and crying at point 5 [78].

#### Unvalidated questionnaire measures

Many of the measures identified in our systematic search have not been psychometrically examined in women with pelvic mesh implants but had been evaluated in other samples (see Table 2). However, we also identified 28 studies using measures or adapted measures that have not been psychometrically evaluated in any samples to-date. Notable examples include Izett-Kay and colleagues [30], who designed a novel questionnaire to examine patient-reported mesh complications requiring the removal of the mesh after a laparoscopic mesh sacrohysteropexy procedure for pelvic organ prolapse. The questionnaire probed the nature and timing of symptoms that led to the diagnosis of a mesh-associated complication. Items referencing pain include an item assessing why the mesh was removed, where respondents can indicate that this was due to chronic pain. Another item examines the problems caused by the mesh complication, where respondents can indicate pain on physical examination, pain during sexual intercourse, pain during physical or daily activities, or pain that is characterised in another way (among other symptoms). In addition, the questionnaire includes an item probing whether the responded is currently under the care of a pain specialist due to mesh-related complications.

In another notable example, Brown and colleagues [79] developed a novel questionnaire to examine knowledge and perceptions of vaginal mesh surgery in women presenting as new patients in a urogynaecology clinic. Within the questionnaire, one item probed whether the respondent had heard that “mesh can cause pain, including with sex”, among other listed complications. The questionnaire also included items probing the extent to which the respondent is concerned about possible surgery with mesh, and the extent to which the respondent would avoid surgery using mesh, based on their current knowledge and perceptions.

Meanwhile, rather than develop a novel questionnaire, Brown [27] adapted the International Consultation on Incontinence Questionnaire-Lower Urinary Tract Symptoms**.** Specifically, Brown [27] substituted the term “urinary problem” for “mesh problem”, to gather data on the extent to which mesh-related symptoms interfered with the everyday lives of seven women with pelvic mesh complications. These data were gathered alongside qualitative interviews (see Table 1).

Finally, nine studies identified in our systematic search reported using the NSF-9. The NSF-9 a 9-item questionnaire that examines multiple aspects of sexual functioning, including sexual desire, frequency of sexual activity, lubrication, orgasm, and sexual satisfaction [80]. One item (Item 8) pertains specifically to pain: “During the past month, how often have you experienced pain in your genitals before, during, or after sexual contact? (Pain = pain, itching, burning, etc.)”. The severity of symptoms is quantified using a five-point Likert scale. The NSF-9 was originally developed in Dutch [81], but has since been translated into English [80]. However, we could not find any psychometric evaluations of the English translation to date, and psychometric data for the Dutch version could not be obtained.

### Conceptual review

Table 2 summarises the components of pain associated with pelvic mesh implants that are covered by the measures identified in the systematic review. Notably, the collapsed 11-point VAS for Pain (which was utilised in the largest number of studies in our systematised search) covers only one component of the construct: pain intensity. By contrast, the fGUPI [46]—reported in one study—appears to provide the broadest coverage of the components, with five of the six components covered. Of all the components, the impact/interference component was covered by the greatest number of measures, with 19 measures covering at least one of the four subcomponents (physical functioning, sexual functioning/relationships, emotional functioning/mental health, and other aspects pertaining to quality of life) and six measures covering all four subcomponents. The timing and intensity components were also covered fairly well, with 16 measures covering each of the components.

Conversely, the patient expectations/beliefs component was only covered by one instrument: the PCS [72], which also fails to fully encapsulate beliefs or expectations regarding pelvic mesh implants specifically. Nevertheless, within the measures which were novel or lacking in psychometric data, Brown and colleagues [79] developed a questionnaire focused specifically on beliefs and expectations about pelvic mesh implants, and these items may hold promise in tapping this component of pain associated with pelvic mesh implants in future work.

The phenomenological qualities component was covered by eight measures. Of these, the MPQ provides the strongest coverage of the component, with 78 different descriptors included within the questionnaire which cover both sensory-discriminative and affective-motivational aspects of pain. Common phenomenological qualities covered by the other questionnaires included sensations of burning, dragging pain, and heaviness (e.g., BFLUTS, fGUPI, ICIQ-VS, PFDI, POP-SS, and P-QOL). These qualities appear to map fairly well onto the phenomenological qualities that have been frequently reported by women with pain associated with pelvic mesh implants (see Table 1), but perhaps miss sensations related to the feeling of a foreign object inside the body (e.g., [82]. In terms of affective-motivational aspects of pain, the BFLUTS, ePAQ-PF, ICIQ-VS, PUF and P-QOL all include questions on the extent to which one’s symptoms cause “bother”, and both the FSFI and PISQ reference avoidance of sexual intercourse due to pain (the FSFI) or due to vaginal “tightness” or “dryness” (the PISQ).

Finally, the location component was covered by 13 measures, however there was some disparity between the bodily locations included in the measures and the bodily locations that have been frequently reported by women with pain associated with pelvic mesh implants (see Table 1). Locations that are particularly important to the construct include the vaginal area, the pelvic area, the buttocks, groin, hips, leg, lower back, and the incision site (see Table 1). However, the majority of the measures identified that mention bodily locations concentrate on the vagina, bladder, lower abdomen and pelvis (e.g., the BFLUTS, fGUPI, FSFI, ICIQ-VS, KHQ, PFDI, and PISQ; see Sect. 3.1.). The symptom severity section of the P-QOL and the POP-SS are very slightly more comprehensive in terms of location, in that some items pertain to the lower back. Notable exceptions to this include the BPI and the MPQ, which both include full body maps which are used to note the locations of pain.

### Psychometric review

Table 3 summarises the evidence of psychometric properties for the questionnaire measures in samples that have included women with pelvic mesh implants. It is notable that none of the measures in this group were specifically designed to capture outcomes from pelvic mesh procedures (see Sect. 3.1), with the majority of the identified measures having been designed to assess symptoms of pelvic organ prolapse, urinary incontinence, or as generic outcome measures (e.g., EQ-5D-5L, SF-36).

**Table 3:** Evidence of the psychometric properties of the included measures in samples of women with pelvic mesh implants

Name of measure	Number of items	Structure of measure	Response scale type	Cronbach α coefficients	Test–retest reliability		Validity
Brief pain inventory	15	2 factors	11-point numeric rating scale, and body map for pain location	–	–		*Concurrent validity*: BPI scores significantly correlated with amount of analgesia used for post-operative pain control in a sample of women undergoing pelvic floor reconstructive surgeries, including mesh procedures [83]
Bristol lower urinary tract symptoms questionnaire-SF	19	3 factors for urinary symptoms, and two additional sections for sexual function and QOL	4-point, 5-point Likert scales	.66–.75 [41]	–		*Sensitivity to change***:** 13 items (including bladder pain) had a statistically significant change post-treatment in a sample of women with tension-free vaginal tape [41]
Electronic personal assessment questionnaire—pelvic floor	132	4 dimensions, 19 domains	4-point Likert scales				*Minimally important difference*: Identified in a sample of women with tension-free vaginal tape [84] *Sensitivity to change*: The EPAQ-PF was responsive to change in health status in a sample of women with tension-free vaginal tape, with the pain items demonstrating acceptable responsiveness statistics [84]. Effect sizes for the urinary pain (0.3), vaginal pain (0.7) and sensation, and dyspareunia (0.6) scales were small-to-moderate
EQ-5D-5L	6	5 unique items	5-point Likert Scale	–	–		*Convergent validity*: Small-to-moderate correlations with PFDI-20 scores in a sample undergoing surgical POP repair using transvaginal mesh [85]
Female sexual function index	19	6 factors	6-point Likert Scale	–	–		*Sensitivity to change*: Small changes in FSFI domain scores in a sample of women with anterior mesh implants for the treatment of cystocele (effect sizes = 0.01–0.2 [86])
King’s health questionnaire	32	3 sections, and 7 QOL domains	3-point and 4-point Likert scales	Symptom severity = .82–.91; QOL domains = .41–.95 [87]		–	*Sensitivity to change*: Moderate to excellent responsiveness in a sample of women undergoing surgery for stress incontinence, including mesh procedures (effect sizes = 0.76 – 2.40 [87]) *Convergent validity*: The role limitation, physical limitation, social limitation, incontinence impact, severity measures, and sleep/energy QOL domains had moderate-to-strong correlations with total PISQ score in a sample of women undergoing surgery for POP/SUI, including mesh procedures [88]
Patient global impression of improvement	1	(Single item)	7-point Likert Scale	–	–		*Minimally important change*: Frequently used as an anchor for the assessment of minimally important change in other measures (e.g., [85, 89])
Pelvic floor distress inventory-20	20	3 scales scored separately, global summary score calculable	5-point Likert Scale	Summary score = .82–.85; Scales = .68–.78 [85]	ICC for summary score = .92–.93 [52, 89]		*Minimally important change*: Identified in samples undergoing surgical treatment for POP (12% vaginal mesh; [89]) and for any pelvic floor dysfunction (40% tension-free vaginal tape [52]) *Sensitivity to change*: Excellent sensitivity to change in samples undergoing surgical treatments for any pelvic floor dysfunction (40% tension-free vaginal tape; effect size = 1.48 [52]), and a sample undergoing surgical POP repair using transvaginal mesh (effect size = 1.26–1.37 [85]) *Convergent validity*: Moderate correlations with the PISQ-12, PIFQ-7, PGI, and EQ-5d [85]. Strong correlation with the PGI 6 months after mesh surgery [89]
Pelvic floor impact questionnaire-7	31	3 scales scored separately, global summary score calculable	4-point Likert scale	Summary score = .95–.96; Scales = .89–.93 [85]	ICC for summary score = .77 [52]		*Minimally important change*: Identified in a sample undergoing surgical treatment for any pelvic floor dysfunction (40% tension-free vaginal tape [52]) *Sensitivity to change*: Moderate sensitivity to change in a sample undergoing surgical treatment for any pelvic floor dysfunction (40% tension-free vaginal tape; effect size = .67 [52]), and a sample undergoing surgical POP repair using transvaginal mesh (effect size = 0.75–0.91 [85]) *Convergent validity*: Weak or non-significant correlations with scores for pelvic organ prolapse severity grade (POP-Q), but strong correlations with PFDI and PGI in a sample undergoing surgical POP repair using transvaginal mesh [85] *Discriminant validity*: Limited evidence [85]
Pelvic organ prolapse–urinary incontinence sexual function questionnaire-12	12	1 factor	5-point Likert scale	.62–.84 [88, 90]	–		*Convergent validity***:** Moderate correlations with the PFDI and PFIQ in a sample of women with transvaginal mesh implants [90] *Sensitivity to change*: Responsive to changes in sexual functioning post-surgery, with moderate effect sizes documented at 3 months and 6 months [90] However, a mixed methods study indicates that while the PISQ-12 may be sensitive to positive impacts of mesh implants, it seems to miss new negative symptoms resulting from the implant surgery [31]
Pelvic organ prolapse symptom score	8	1 factor	5-point Likert Scale	.82–.83 [56]	69% score agreement over *M* = 36 days [57]		*Known groups validity*: POP-SS scores were significantly higher in women due to undergo mesh implant, versus women with conservative management or no management [56] *Minimally important difference***:** Identified in samples of women undergoing prolapse surgery with and without mesh [57]. Previously reported differences from intervention [56] exceed minimally important change *Sensitivity to change*: Significant differences in scores before and after mesh surgery, and this difference was greater than the difference observed in a conservatively managed group [56]
Prolapse quality of life questionnaire	38	9 QOL domains; total score calculable	4-point Likert scale	–	–		*Minimally important difference*: weak evidence – indicated using the half a standard deviation model [91] in a sample undergoing vaginal mesh implantation for cystocele [82] *Sensitivity to change*: Weak evidence – significant improvements were observed for all nine domains of the questionnaire after vaginal mesh surgery [82]. The differences were greater than the minimally important difference criterion outlined above
Short-form health survey-36	36	2 higher-order factors and 8 lower-order factors	Range of 3-point, 5-point, and 6-point Likert Scales and binary (yes/no) options	–	–		*Sensitivity to change*: Only the social functioning scale demonstrated moderate responsiveness (effect size = .75), the remaining scales (including the bodily pain scale) were not responsive to change in samples undergoing surgical treatments for any pelvic floor dysfunction (40% tension-free vaginal tape [52]) *Concurrent validity*: The bodily pain and physical functioning scales of the SF-36 had strong correlations with the Activities Assessment Scale (a measure of post-operative functional activity) in a sample of participants with tension-free vaginal tape at baseline, 2 weeks, and 6 months post-surgery [92]

Overall, there was limited evidence of psychometric evaluation in samples of women with pelvic mesh implants. Of all the measures identified, the PFDI-20, PFIQ-7, POP-SS, and PISQ-12 appear to have appropriate properties for use in samples of women with pelvic mesh implants. However, it should be noted that the investigations of the psychometric properties for each of these measures (the PFDI-20, PFIQ-7, POP-SS and PISQ-12) has been focused on the overall change in pelvic organ prolapse or urinary incontinence symptoms following pelvic mesh implant surgery. To our knowledge there are no studies specifically examining the psychometric properties of these measures in women with pain associated with a pelvic mesh implant. Therefore, it is unclear at present whether any of the measures included in this review are psychometrically appropriate for the assessment of pain related to pelvic mesh implants.

## Discussion

The aim of this review was to examine ways in which pain associated with pelvic mesh implants has been measured to date, with a specific focus on the questionnaire measures that have been utilised. Overall, we identified 28 different assessments of pain associated with pelvic mesh implants, and 61% of the included studies used more than one measurement tool. The questionnaire measures that we identified were grouped into three categories: measures designed to assess urological and or/pelvic symptoms, generic measures (including measures validated across numerous pain disorders), and unvalidated measures (including novel measures, or novel adaptations of measures). Crucially, we were unable to find any validated measures that were specifically designed to assess pain associated with pelvic mesh implants.

In our preliminary narrative review, we integrated existing qualitative and theoretical accounts of the pain associated with pelvic mesh implants, which this informed the development of a multidimensional construct definition. We used this construct definition to assess the comprehensiveness of the questionnaire measures identified in the scoping review. While many of the measures identified in this review provided coverage of the timing, intensity, and impact/interference components of pain associated with pelvic mesh implants, there were three components that were not captured well (i.e., the phenomenological qualities, location, and expectation/beliefs components). This is important because each of the three underrepresented components of the construct might have implications for targeting patient interventions.

First, regarding the phenomenological qualities component, a questionnaire with sufficient coverage (e.g., the expanded and revised version of the Short-form McGill Pain Questionnaire [93]) might enable practitioners to identify individuals with neuropathic pain and to suggest specific drug treatments. Second, the location component has important implications for pain aetiology and therapeutic interventions. For example, postoperative pain present in areas other than the location of original mesh placement could indicate mesh migration. Finally, regarding the expectations/beliefs component, there is a growing understanding that pain experiences are influenced by top-down factors such as expectations and beliefs. For example, research has indicated that when preoperative expectations for the surgical treatment of pelvic floor disorders were not met, women judged suboptimal outcomes as more serious adverse events [94], and this trend is reflected in the qualitative experiences of women with complications from pelvic mesh implants (see Table 1, see also [27, 29–32]). Furthermore, participants with a high level of pain catastrophisation have been found to have higher levels of pain intensity [95]. Accordingly, formal assessment of patient expectations and beliefs is necessary for tailoring interventions, and can help to identify patients who would benefit from psychosocial interventions, such as educational or counselling sessions.

We also considered evidence of the psychometric properties of the included measures in samples of women with pelvic mesh implants. Overall, we were able to identify limited evidence indicating that the currently-available tools are appropriate for use in samples of women with pelvic mesh implants. However, the investigations of the psychometric properties were focused on the overall change in pelvic organ prolapse or urinary incontinence symptoms following pelvic mesh implant surgery. Thus, at present it is not clear whether any of the measures included in this review are psychometrically appropriate for the assessment of pain related to pelvic mesh implants.

Within the measures that are unvalidated, the work of Izett-Kay and colleagues [30], Brown and colleagues [79], and Brown and colleagues [27] to develop or adapt measures specifically for women with pelvic-mesh implants is promising. It is possible that items from these measures could be used to inform the development a condition-specific patient reported outcome measure (PROM) which captures the full range of components from the overall construct (i.e., pain associated with pelvic mesh implants). In particular, the questionnaire developed by Brown and Colleagues [79], which covered knowledge and perceptions of vaginal mesh surgery, gave the most comprehensive coverage of the expectations/beliefs component of the construct.

A condition-specific PROM would be particularly useful for patients undergoing complex pelvic surgery to remove pelvic mesh implants, who require adequate perioperative assessment and counselling. Such surgery can be complex and can, in some instances, lead to damage to surrounding organs and tissue and result in recurrent prolapse and incontinence symptoms and ongoing pain (e.g., [4, 13]). A condition-specific PROM would also be useful for future research into therapeutic interventions for pain associated with pelvic mesh implants. For example, at present there is limited research on the effectiveness of mesh removal procedures for pain associated with pelvic mesh implants, and efficacy estimates vary greatly, possibly as a function of the measurement tool that is used [9–13, 22, 25, 96].

One limitation of the present work is that our construct development phase relied on existing qualitative and theoretical accounts of the construct, in addition to the knowledge and experience of the authors, and feedback from a PPI panel of ten women with pain related to pelvic mesh implants. As a direction for future research, we suggest that further formal qualitative research is conducted to better understand the experiences of pain assessment and management in women with pain associated with pelvic mesh implants. This qualitative work could inform the development of a condition-specific PROM aimed at assessing mesh-related pain, or assessing the outcomes of interventions for mesh-related complications. An additional limitation of the present work is that this review only included questionnaires used in English-speaking populations, which is an additional issue to consider in future research, as participants from different cultural backgrounds might conceptualise pain associated with pelvic mesh implants differently (e.g., it is feasible that different groups may place more emphasis on one particular domain, such as impact/interference). A final limitation was that we employed a search strategy whereby one reviewer completed article screening and study selection, and a second author screened a random 10% of the articles.

### Conclusion

Overall, we reviewed the ways in which pain associated with pelvic mesh implants has been measured to date, focusing on the questionnaire measures that have been utilised. We were unable to find any validated measures that were specifically designed to assess pain associated with pelvic mesh implants. Our conceptual review indicated that most of the existing measures provided inadequate coverage of the phenomenological qualities, location, and expectation/beliefs components of pain associated with pelvic mesh implants. Furthermore, we were able to identify limited evidence indicating that the existing measures are appropriate for use in samples of women with pelvic mesh implants. We recommend future development of a condition-specific PROM to address these issues.

## Data Availability

The dataset that was generated and analysed for the systematised review is available in the Figshare repository, which can be accessed here: https://figshare.com/s/88d23a55f7cad5833856.

## Abbreviations

BFLUTS: Bristol female lower urinary tract symptoms questionnaire
BPI: Brief pain inventory
CRADI: The colorectal-anal distress inventory
CRAIQ: Colorectal-anal impact questionnaire
ePAQ-PF: Electronic personal assessment questionnaire—pelvic floor
fGUPI: Female genitourinary pain index
FSFI: Female sexual function index
ICC: Intraclass correlation coefficients
ICIQ-VS: International Consultation on incontinence questionnaire-vaginal symptoms
KHQ: King’s health questionnaire
MPQ: McGill pain questionnaire
NRS: Numeric rating scale
PCS: Pain catastrophizing scale
PFDI: Pelvic floor distress inventory
PFIQ: Pelvic floor impact questionnaire
PGI: Patient global impression of improvement
PISQ: Pelvic organ prolapse–urinary incontinence sexual function questionnaire
POPDI: Pelvic organ prolapse distress inventory
POPIQ: Pelvic organ prolapse impact questionnaire
POP-SS: Pelvic organ prolapse symptom score
PPI: Patient and public involvement
P-QOL: Prolapse quality of life questionnaire
PROM: Patient reported outcome measure
PUF: Pelvic pain and urgency/frequency patient symptom
SF-36: Short-form health survey
UDI: Urinary distress inventory
UIQ: Urinary impact questionnaire
VAS: Visual analogue scale
WBS: Wong–Baker FACES scale
